# Effect of Foliarly Applied Orange Carbon Dots on Grain Yield and Quality in Maize Hybrids and Inbred Lines

**DOI:** 10.3390/plants15010008

**Published:** 2025-12-19

**Authors:** Ivana Milenković, Zoran Čamdžija, Slađana Žilić, Milan Borišev, Slađana Z. Spasić, Ksenija Radotić

**Affiliations:** 1 Institute for Multidisciplinary Research, National Institute of the Republic of Serbia, University of Belgrade, Kneza Višeslava 1, 11030 Belgrade, Serbia; sladjana@imsi.bg.ac.rs; 2 Maize Research Institute, Zemun Polje, Slobodana Bajića 1, 11185 Belgrade, Serbia; zcamdzija@mrizp.rs (Z.Č.); szilic@mrizp.rs (S.Ž.); 3 Department of Biology and Ecology, Faculty of Sciences, University of Novi Sad, 21000 Novi Sad, Serbia; milan.borisev@dbe.uns.ac.rs

**Keywords:** carbon dots, grain yield, photosynthesis, *Zea mays* L., elevated carbon dioxide

## Abstract

Maize is a key staple cereal, with its cultivation improved through genetics, denser planting, and greater fertilizer use. However, little is known about the effects of nanomaterials on maize’s grain quality. This study evaluated the effect of the foliar application of orange carbon dots (o-CDs) on maize’s growth, grain yield, and quality under typical field conditions. Two ZP maize hybrids and their inbred lines were tested. The results showed a gradual increase in grain yield for the hybrids, particularly ZP 4567, which responded significantly to a 5 mg/L treatment. Increased starch content was observed in both the hybrid ZP 4567 and the inbred line L56 L026 following treatment with o-CDs at concentrations of 1 mg/L and 5 mg/L. The significant increase in oil content was observed in inbred line L56 L026. Photosynthetic parameters and pigments were elevated in both hybrids after treatments, although the antioxidative capacity remained unchanged. The findings suggest that o-CDs positively influence grain yield and quality by enhancing photosynthesis and increasing the accumulation of key biochemical compounds. This study provides novel insights into the application of carbon nanoparticles in sustainable crop production.

## 1. Introduction

Maize is one of the world’s leading staple cereals. It is a multi-purpose crop, primarily used as a feed and food crop but also used for industrial purposes such as in alcohol or syrup production. The sugar derived from maize is one of the main sweeteners in soft drinks. Increasingly, ethanol made from maize is being used as an additive in petrol [1].

Maize is the primary agricultural crop, along with wheat, grown in the Republic of Serbia, with approximately 750,000–800,000 hectares planted annually. Among the harvested areas under cereals, maize dominates with 53% [2]. According to FAOSTAT 2020, Serbia is ranked 19th globally for corn production, with an average five-year grain yield (2016–2020) of 7.9 million tons. The increase in grain yield is mainly attributed to breeding efforts (around 60%) resulting in genetic improvements in cultivated maize hybrids [3].

Breeding programs typically consist of breeding cycles. Each new breeding cycle leads to the development of fresh maize varieties ready for cultivation using innovative agricultural methods [4]. The stable and high grain yield performance of maize F1 hybrids must be supported by stable and high-yield seed production. Maize seed production in Serbia has a strong tradition, particularly in the context of the significant maize production worldwide, with great attention being devoted to producing and improving seed quality in scientific institutes [5,6,7].

Agricultural practices in maize cultivation have evolved alongside advancements in maize genetics. These practices are mainly characterized by higher planting densities, increased use of fertilizers, and plant protection measures. The high grain yield production of maize today relies heavily on the use of fertilizers, particularly nitrogen fertilizers [8]. Carbon-based materials have been used to improve photosynthesis and yield in maize [9]. More modern agricultural practices may involve the use of foliar fertilizers. Since leaf tissue has a similar morphological structure to root tissue, plants can absorb dissolved minerals [10]. With the development of the nanotechnology field, engineered nanomaterials have been studied as potential stimulators of different characteristics of agricultural plants such as growth and grain yield enhancement [11]. Carbon-based nanomaterials, “carbon dots” (CDs), due to their many good physicochemical properties, have been studied for possible applications in various fields due to their non-toxicity [12].

Crop yield increases with an enhancement in leaf photosynthesis. Plants do not use the maximal capacity of their photosynthetic apparatus [13]. In the previous studies, we demonstrated an increase in their photosynthetic parameters without toxic effects on the maize plants, using a type of CD, orange CDs (o-CDs), in hydroponics [14]. The effect was more pronounced when it was sprayed on leaves compared to its addition via a medium. In a small field study, o-CDs were foliarly applied on the maize (*Zea mays* L.) and green beans (*Phaseolus vulgaris* L.) [15]. Photosynthesis was enhanced in both plant species, and green beans showed an increased fruit yield.

This study investigated the effect of the foliar application of orange carbon dots (o-CDs) on maize growth, grain yield, and composition, when applied over a larger cultivation area in the typical environmental conditions of field-grown corn. These conditions mirror the standard application procedures used for conventional fertilizers in agriculture, making this experiment a step toward the practical use of o-CDs as growth and yield stimulators. Additionally, there is a lack of information in the literature about the effects of applying carbon nanoparticles on maize’s composition and quality. We hypothesized that the foliar application of o-CDs would enhance photosynthetic performance and increase grain yield in both maize hybrids and their corresponding inbred lines under standard field conditions, compared with the untreated controls. We further hypothesized that o-CD treatment would modify key grain quality parameters, including protein, starch, and oil content. Overall, we expected that, under typical agricultural conditions, o-CDs would function as effective stimulants of photosynthesis, leading to increased grain yields and improved grain composition. Two ZP maize hybrids and their corresponding maternal inbred lines were selected to evaluate the potential increase in grain yield for commercial corn production and seed production.

## 2. Material and Methods

### 2.1. Synthesis of o-CDs

O-CDs were obtained from the Department of Chemistry, University of Miami. The processes of synthesizing and characterizing o-CDs were previously described by Zhou et al. (2018) [16]. In summary, the o-CDs were created using a molar ratio of 1:25 citric acid and o-phenylenediamine, which were dissolved in 10 mL of deionized water (DI). The mixture was then subjected to ultrasonication for 1 h at 42 kHz under the protection of argon gas. This resulted in an orange aqueous dispersion that exhibited yellow photoluminescence under UV light (365 nm). To obtain the orange powder, unreacted o-phenylenediamine was filtered out using an ice bath, followed by size exclusion chromatography (SEC) using GE Healthcare Sephacryl S-300 matrix (Uppsala, Sweden) to remove small fluorophores, and, finally, the product was lyophilized for 2 days. The previously reported characterization showed that the obtained o-CDs are spherical in shape, with a narrowly distributed size within 1–4 nm (mean diameter of 2 nm), and the zeta potential indicated that they carry a negative charge, with a potential value of −12.2 mV [16]. The absorption, emission spectra, and fluorescence quantum yield data can be found in the previous publication [14].

### 2.2. Treatment of Maize with o-CDs

In this study, two ZP maize hybrids were used: ZP 4567 (FAO 400) and ZP 6263 (FAO 600), along with their maternal components L56 L026 and L76 B044, respectively. These hybrids are part of the market-approved ZP varieties of the most important maturity groups harvested in the Republic of Serbia—FAO 400 and FAO 600. This field experiment was conducted over two consecutive seasons (2021 and 2022) at different locations. The present manuscript reports findings from the second year (2022), chosen due to enhanced treatment optimization and consistent climatic conditions. The meteorological conditions for the year 2022 include an average air pressure of 1000.05 mb, an average air temperature of 22.08 °C, an average relative humidity 60.5%, and an average rainfall of 49.78 mm for the period of maize cultivation (middle April to middle September), according to the Republic Hydrometeorological Service of Serbia.

The trial itself was set on high-quality soil with the following parameters: pH (KCl) = 7.30; pH (H_2_O) = 8.21; CaCO_3_ = 8.40%; humus = 2.50%; total nitrogen = 0.186%; P_2_O_5_ = 34.2 mg/100 g; and K_2_O = 28.2 mg/100 g. According to the soil classification IUSS Working Group 2014, 2022, such soil represents Haplic Chernozem. The trial took place at the Maize Research Station (MRIZP) in Zemun Polje in 2022, with each hybrid and maternal component planted at a density of 66,667 plants/ha in two repetitions. Each plot consisted of four rows, with an inter-row space of 0.75 m and a row length of 5 m, resulting in a total area of 15 m^2^ and 100 plants planted per plot. To prevent potential wind damage to the plants, each plot was surrounded by four protection rows. Planting and harvesting were carried out using Wintersteiger Dual Disc.

The 15 L of aqueous solutions for each o-CD concentration (1 and 5 mg/L) were prepared in tap water, simulating the application of nanoparticles by farmers. Since Milenković et al. (2021) [14] previously reported that 1 and 5 mg/L of o-CDs increased the photosynthetic parameters in maize plants after foliar application, these o-CD concentrations were chosen for the maize treatment in this study. The o-CDs were applied three times to both plant species at equal intervals during the vegetative growth stage until flowering. This was performed by spraying the leaves with 1 or 5 mg/L of the o-CD aqueous solutions. The treatments were carried out on dry days, just before sunset, to avoid direct sunlight and minimize the risk of undesired wind flow. Two 15 L backpack sprayers were used, each for a different concentration. The complete plot area with the corresponding maize plants was treated. The first application took place in the V3 vegetation period of maize, with each variety having three fully developed leaves (17 May 2022), and then repeated two more times in two-week intervals (1 June 2022 and 15 June 2022). For comparison, there was a control without o-CD treatment for both hybrids (ZP 4567 and ZP 6263) and maternal lines (L56 L026 and L76 B044).

After the treatment period, the leaves were collected, frozen in liquid nitrogen, and kept at −80 °C until the determination of total phenolic content (TPC), antioxidative activity (TAA), and photosynthetic pigments (chlorophyll and carotenoid content).

### 2.3. Grain Yield Determination

The obtained results were recalculated in t/ha at moisture level of 14% according to (Equation (1)). Based on Article 6, Paragraph 1 of the Law on Technical Requirements for Products and Conformity Assessment, the storage of maize grain has to be conducted with a moisture content of 14%; therefore, the obtained raw yield results from the field were recalculated in t/ha with the level moisture content to 14% by using following formula [17]:

(1) Y=0.6667B(100−E)/(100−14)

*Y*—grain yield in t/ha with 14% moisture;

*B*—raw grain yield of tested genotype per plot (15 m^2^) in moment of harvest;

*E*—raw moisture content of genotype in moment of harvest;

0.6667—coefficient to calculate grain yield in units t/ha.

### 2.4. Analysis of Basic Chemical Composition of Maize Grain

The oil content was determined according to the Soxhlet standard method (AOAC, 2000) [18]. The Soxhlet method adapted for BÜCHI FatExtractor system (BÜCHI Labortechnik, Flawil, Switzerland) is a gravimetric solid-liquid extraction technique used to determine the oil or fat content from solid samples by repeatedly washing them with a non-polar organic solvent. This method utilizes continuous solvent recycling through vaporization, condensation, extraction, and siphoning processes to effectively dissolve and separate lipids from the sample. The protein content was determined indirectly through the content of total nitrogen multiplied by a 6.25 conversion factor according to the standard micro-Kjeldahl method (AOAC, 1990) [19], using the AutoKjeldahl distillation unit K–350 and speed digester K–439 (BÜCHI Labortechnik, Flawil, Switzerland). The process includes automated digestion with concentrated sulfuric acid, steam distillation, and titration to quantify ammonia. Ewers’ polarimetric method (ISO 10520, 1 September 1997) [20] was used for starch content determination. In this method, the starch is released from the sample via boiling in diluted hydrochloric acid (HCl). This procedure effectively gelatinizes the starch granules and simultaneously hydrolyzes starch to glucose in a single step. The acid also helps break down the endosperm tissue, which ensures the complete release of the starch granules from the protein matrix. Substances that may interfere with the measurement are removed by filtration/clarification, and then the glucose concentration is determined by measuring the angle of polarization or optical rotation. Polarimeter UniPol L 2020 (Schmidt + Haensch GmbH and Co., Berlin, Germany) was used to determine the optical activity and measure the starch content. The content of crude cellulose was determined according to the Weende method (AACC, 1999) [21] using the Fibertec system FOSS 2010 Hot Extractor (FOSS Tecator, Hoeganaes, Sweden). The Weende crude fiber method involves boiling the defatted samples in dilute sulfuric acid and potassium hydroxide, dissolving non-fiber components, and leaving primarily cellulose and lignin. The process includes drying the maize flour, acid digestion, base digestion, filtration, drying the residue, ashing, and a final weighing, to calculate crude fiber as a percentage of the original sample weight. All the results are given as the percentage per dry matter (d.m.). 

### 2.5. Measurements of Photosynthetic Parameters

The following parameters were measured to indicate photosynthesis and water use: PR (μmol CO_2_ m^−2^s^−1^) and TR (mmol H_2_O m^−2^s^−1^), using the LC pro + portable photosynthesis system (ADC Bioscientific Ltd., Hoddesdon, UK). WUE (μmol CO_2_ mmol^−1^ H_2_O) was calculated as a ratio of PR/TR. The measurements were conducted with four replicates on five different plants per treatment. The air supply unit provided a constant ambient airflow rate of 100 μmol s^−1^. The temperature, humidity, and CO_2_ concentrations were at ambient levels. The photosynthetic rate (PR) and transpiration rate (TR), along with their ratio in the form of water use efficiency (WUE = PR/TR), were assessed 2 days following each o-CD treatment. The photosynthetic parameters were measured two days after each treatment, on May 19th and June 3rd, for both used o-CDs concentrations and for the control plants.

### 2.6. Determination of the Photosynthetic Pigments

The frozen leaves were extracted using 80% acetone (1:10 *w*/*v*). The resulting extracts were centrifuged at 9.3× *g* at room temperature, and the precipitates were then re-extracted with acetone. The supernatants were combined and diluted with 80% acetone. The absorbance was measured at 663, 646, and 470 nm. The concentrations of chlorophyll (Chla, and Chlb), and carotenoid content (Car) were determined using a spectrophotometer (2501 PC spectrophotometer, Shimadzu, Kyoto, Japan), and the values were expressed in μg of pigment per g of fresh weight [22].

### 2.7. Determination of Total Phenolic Content (TPC)

Phenolic extracts were obtained from the leaves after three foliar treatments. They were separately homogenized in a mortar with liquid nitrogen. The homogenates were then resuspended in 80% methanol in a 1:10 (m/V) ratio and stirred at room temperature for 60 min. The extracts were centrifuged for 5 min at 9.3× *g*, and the phenolics were obtained in the supernatant.

For the determination of total phenolic content (TPC), the Folin–Ciocalteu’s spectrophotometric procedure [23] was used. Phenolic extracts were mixed with Folin–Ciocalteu reagent in a total volume of 1 mL. After 3 min, sodium carbonate solution was added, and the mixture was incubated at 25 °C for 60 min. A standard curve (0.1–2.0 mM) was constructed using gallic acid. Absorbance was read at 724 nm using a 2501 PC spectrophotometer (Shimadzu, Kyoto, Japan), and the results were expressed as μmol of gallic acid equivalents per gram of fresh weight.

### 2.8. Determination of Total Antioxidant Activity (TAA)

The total antioxidant activity (TAA) of the samples was measured using the ABTS/HRP endpoint method, as per a modified procedure described in the literature [24]. In summary, the reaction mixture consisted of 2 mM ABTS, 15 μM H_2_O_2_, 0.25 μM HRP, and 20 μL of 80% methanol extract from the samples in a 50 mM potassium phosphate buffer at pH 7.5, in a total volume of 1 mL. The assay was conducted in five replicates per treatment, at 25 °C. The reaction was monitored by spectrophotometry at 730 nm (Shimadzu, Kyoto, Japan) until a steady absorbance was achieved, due to the formation of ABTS radical (ABTS + in the reaction with HRP). Following the addition of methanolic plant extracts, the decrease in absorbance due to ABTS + depletion was used to calculate TAA from the standard curve obtained with ascorbic acid (0.1–1 mM) as a universal antioxidant. The TAA was expressed as a μmol value of the ascorbic acid equivalents per gram of fresh weight.

### 2.9. Statistical Analysis

Exploratory and data analyses were performed using IBM SPSS Statistics 25 software (IBM Corporation, New York, NY, USA). The raw data, TPC, TAA, and pigments’ concentration (Chla, Chlb, and Car) in maize leaves after foliar solution treatments, were used as input variables. The number of observations was *n* = 10 for each group.

The one-way analysis of variance (ANOVA) for independent samples was applied to test the differences in TPC, TAA, and pigments’ concentration measured in maize leaves under different treatments inside the same maize variety. Post-hoc inter-group comparisons of variables (between different treatments and control) were performed by LSD post-hoc tests at the level of significance α = 0.05.

The one-way ANOVA test was applied to evaluate the effect of o-CD concentration on three measured properties (PR, TR, and WUE) at two-time points, a few days from the treatment. The number of observations was *n* = 10 for each group. ANOVA tests were followed by an additional post-hoc Tamhane test with the significance level α = 0.05.

In order to assess the impact and efficiency of the foliar application of different concentrations of o-CDs on the grain yield and grain quality parameters (protein, starch, oil, and cellulose) in maize varieties ZP and their maternal lines, the one-way ANOVA and subsequent LSD test (α = 0.05) were also conducted.

## 3. Results

**Figure 1:**
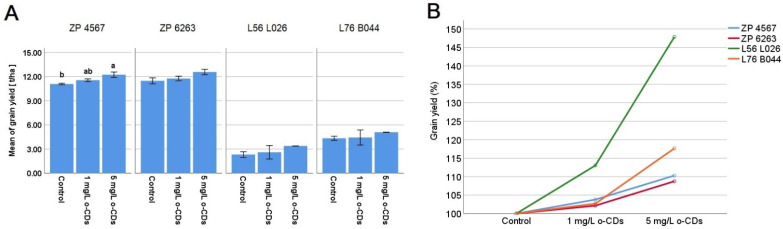
Grain yield of maize hybrids (ZP 4567 and ZP 6263) and maternal lines (L56 L026 and L76 B044) when cultivated under control conditions and foliar treated with two different concentrations of o-CDs solution. (**A**) Mean values (t/ha ± SE). (**B**) Percentage of grain yield increase normalized to 100% of each respective control. ^ab^ indicates a significant difference between treatments inside the same maize variety, at the level of the significance α = 0.05.

The mean grain yield [t/ha] is shown in Figure 1A for the cultivated maize hybrids ZP 4567 and ZP 6263, along with their maternal components, L56 L026 and L76 B044, under foliar application of two different concentrations of o-CDs compared to their respective controls. The gradual increase in grain yield observed for the ZP hybrids and maternal lines under both treatments is clearly visible in their percentage increase over each respective control (Figure 1B). Notably, ZP 4567 produced a grain yield of 12.23 t/ha, demonstrating a statistically significant response to foliar treatment at a concentration of 5 mg/L. The other hybrid ZP 6263 and maternal lines followed a similar pattern, with both treatments leading to higher grain yields for L76 B044 and L56 L026. The highest yields were recorded at the 5 mg/L concentration with L56 L026 yielding 3.385 t/ha and L76B044 reaching 5.095 t/ha. However, statistical significance was not proved in the average yield increase for either maternal line, due to the high variability in the data, despite a relative yield increase exceeding 47.85% in L56 L026 and 17.65% in L76 B044.

The effect of two o-CDs concentrations on the different maize hybrids and their maternal lines is shown in Table 1. A significant increase in starch content was observed in maize hybrid ZP 4567 for both treatments. Contrary to the hybrids, the obtained results showed a reduced starch content after the treatment with 1 mg/L of o-CDs for both maternal lines and after 5 mg/L of o-CDs in L56 L026 maternal line, while the 5 mg/L concentration treatment showed increased starch content for the inbred L76 B044. The only significant increase in oil content was observed in inbred L56 L026 after the treatment with 5 mg/L. No significant changes or pattern of o-CDs’ influence was detected in other three tested varieties, although a change in the oil content ranging from 3.87 to 4.51 was also observed in inbred L56 L026, which was in a range of around 1%. The o-CDs solutions (1 and 5 mg/L) did not have a statistically significant effect on the protein content in the grain of the hybrids, despite both concentrations affecting the protein content reduction in the line L56 L026 and L76 B044 grain by about 12 and 8%, and 7 and 4%, respectively. The effect of different o-CD concentrations was also not statistically significant in cellulose content in any tested hybrids or lines.

**Figure 2:**
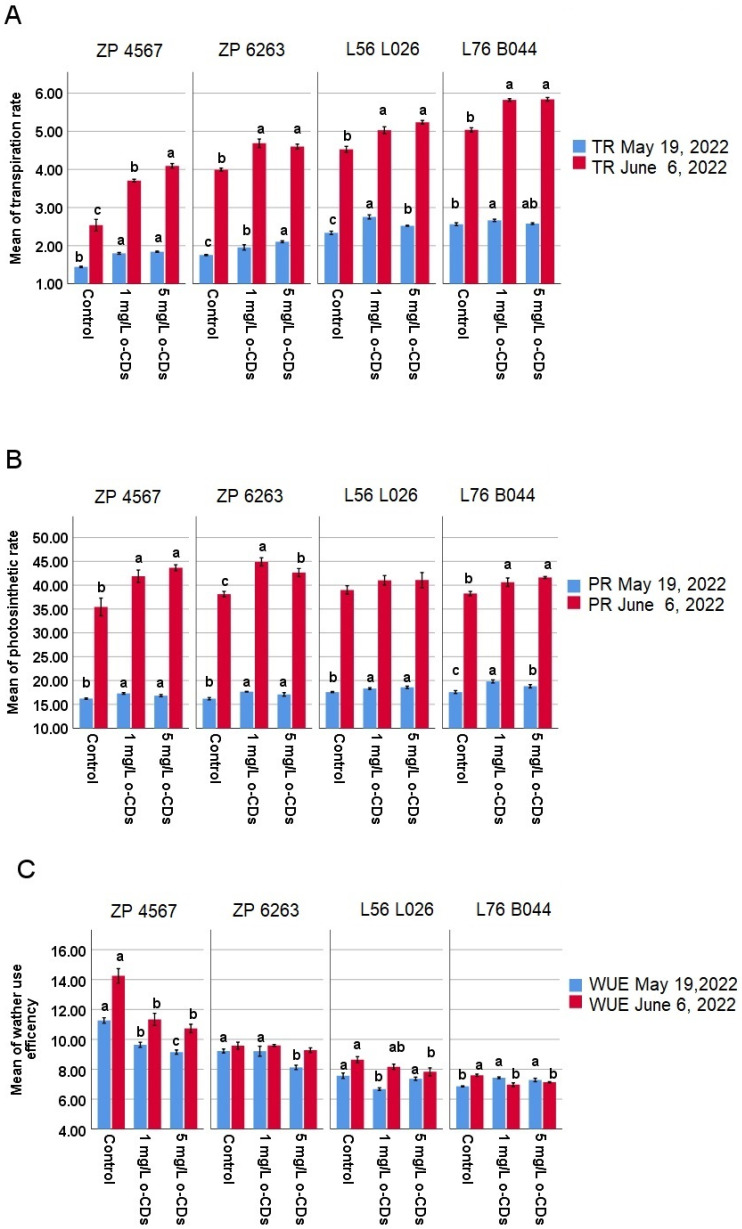
Mean values (±SE) of (**A**) Transpiration rate (TR; mmol H_2_O·m^−2^·s^−1^), (**B**) Photosynthetic rate (PR; μmol CO_2_·m^−2^·s^−1^), and (**C**) Water use efficiency (WUE; μmol CO_2_·mmol^−1^ H_2_O) measured in four maize varieties at the two-time points (19 May 2022 and 3 June 2022). ^abc^ indicates a significant difference between treatments inside the same maize variety and the same time point (blue and red), at the level of the significance of α = 0.05.

An increase in all photosynthetic parameters was observed in both the maize hybrids and their maternal lines after both treatment time points and for both o-CDs concentrations (Figure 2). The photosynthetic rate significantly increased across all genotypes in response to both o-CDs concentrations (Figure 2B), with exception of L56 L026 in the second time point. Correspondingly, the transpiration rate was also enhanced by o-CDs treatment, although one value (the effect of 5 mg/L of o-CDs) for L76 B044 was not statistically significant (Figure 2A). When evaluating water use efficiency (WUE), a generally beneficial reduction due to o-CDs treatment was observed (Figure 2C). Specifically, WUE was significantly reduced in ZP 4567 at both time points and L76 B044 at the second time point. In ZP 6263, this effect was noted only with the 5 mg/L o-CDs application at the first time point while in L56 L026, WUE was reduced at the first time point with 1 mg/L and at the second time point with 5 mg/L.

**Figure 3:**
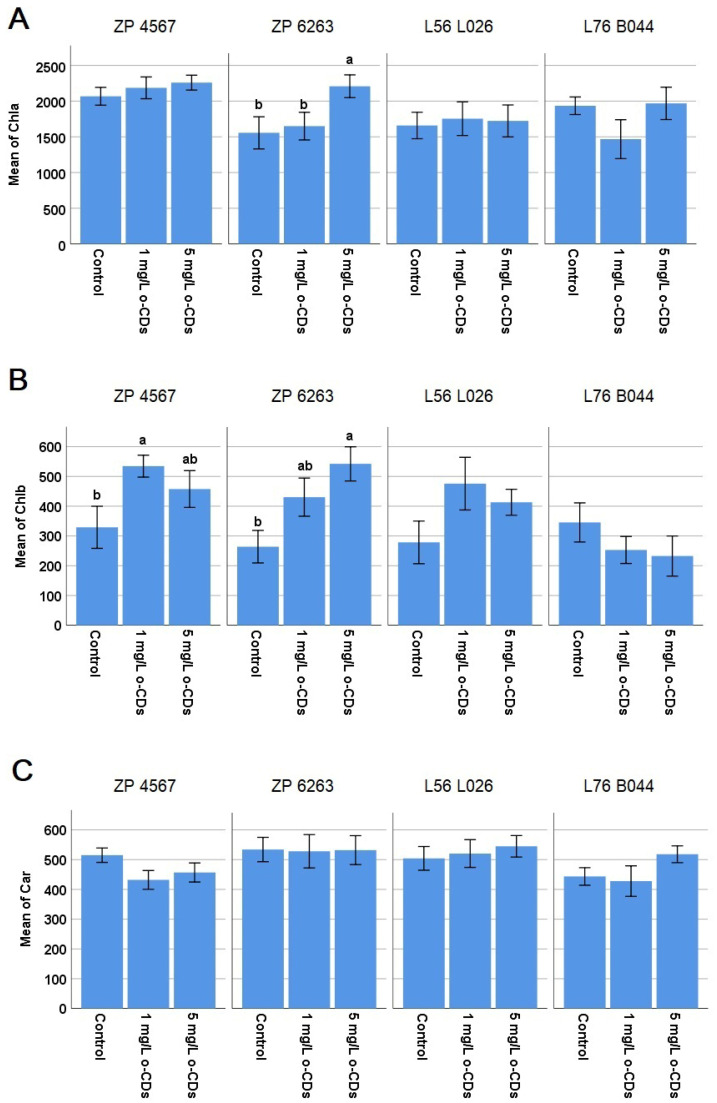
Mean values (±SE) of the content of photosynthetic pigments (**A**) chlorophyll a (Chla; μg/gFW), (**B**) chlorophyll b (Chlb; μg/gFW), and (**C**) carotenoids (Car; μg/gFW) in the maize leaves after foliar treatments with o-CDs solution. ^ab^ indicates a significant difference between treatments inside the same maize variety, at the level of the significance of α = 0.05.

The effect of different o-CDs concentrations on photosynthetic pigments was presented in Figure 3. There was the significant increase in Chlb in ZP 4567 hybrid after 1 mg/L of o-CDs and in Chla and Chlb in the hybrid ZP 6263 after 5 mg/L of o-CDs (Figure 3A,B). The carotenoid content was not significantly changed in any of tested maize varieties. The photosynthetic pigments were not significantly changed in both maternal lines compared to the control after the treatment with any of the two o-CDs concentrations (Figure 3).

**Figure 4:**
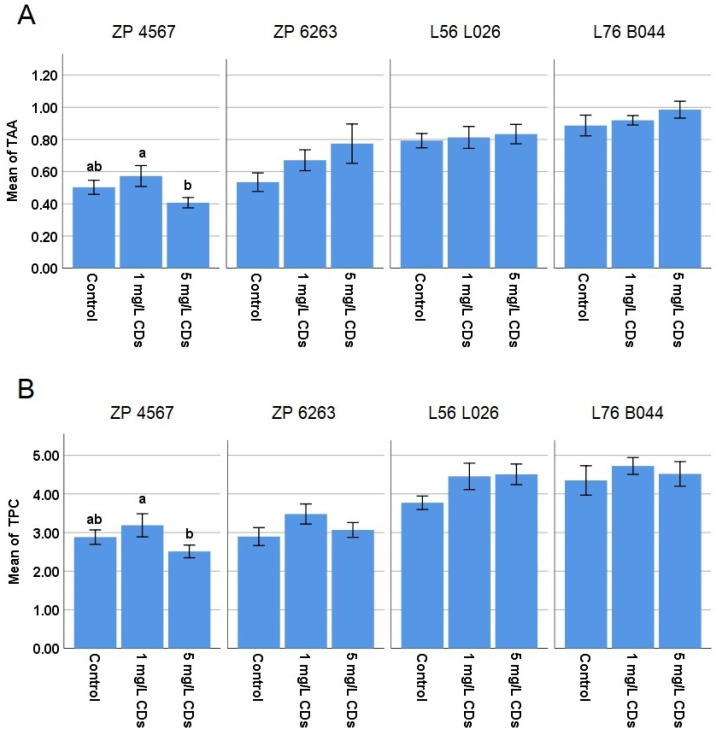
Mean values (±SE) of (**A**) total antioxidative activity (TAA) expressed as equivalent of ascorbic acid/fresh weight (μmol/g) and (**B**) total phenolic content (TPC) expressed as equivalent of gallic acid/fresh weight (μmol/g) in the maize leaves after foliar treatments with o-CDs. ^ab^ indicates a significant difference between treatments inside the same maize variety, at the level of the significance of α = 0.05.

TAA and TPC (Figure 4) were not significantly changed compared to the control for any of the hybrids and maternal lines. There was only a difference between the two treatments in TAA and TPC in the case of the ZP 4567 hybrid. These results indicate that the treatment did not change the antioxidative status of the plants, which is a positive indication for the application of o–CDs as a nanofertilizer.

## 4. Discussion

Due to the constantly growing world population, the global demand for food and agricultural products continues to increase. Therefore, increasing crop yield without compromising fruit quality and causing stress to the plant is of great importance in agriculture. O-CDs have great potential for applications as nanofertilizers due to their ability to increase the efficiency of photosynthesis in maize and green beans [14,15,25]. Therefore, this study provides a significant contribution to understanding the impact of nanoparticles when used as fertilizers.

The results of a gradual increase in grain yield (Figure 1) for the cultivated maize hybrids (ZP 4567 and ZP 6263) and their maternal components (L56 L026 and L76 B044) after foliar application of two different concentrations of o-CDs suggest a clear stimulatory effect relative to control in all hybrids. Both the maize hybrids and their maternal lines followed a similar dose–response pattern, which was only statistically significant in ZP 4567 at a concentration of 5 mg/L. The literature data are in accordance with our results, confirming that carbon dots increased the grain yield in rice by around 13%, using the same mechanism as in our study, through enhancing leaf photosynthesis [26]. Also, increased yields of 15–24.5% after CDs’ application was shown in maize [27].

Carbohydrates, oil, and proteins are major biochemical components of grains, contributing to their nutritional value and functional properties. They are differently affected after the treatment of different maize hybrids and their maternal lines with two o-CDs concentrations, 1 and 5 mg/L (Table 1). Wang et al. (2021) [27] showed that the continuous spraying of CDs (50 mg/L) during the vegetative growth stage of maize can increase carbohydrate accumulation during the reproductive growth stage, leading to the transport of more carbohydrates from leaves to grains, thereby promoting grain filling and increasing yield. This is in accordance with the increase in starch content in ZP 4567 after the treatment with both o-CD concentrations in this study. Furthermore, positive correlations were found between starch and grain yield [28,29], so the influence of o-CDs on one measured trait should impact other traits as well. However, the cellulose content was not changed in this research. The oil content was increased only in the inbred line L56 L026 after the treatment with higher o-CD concentration. Oil and starch are accumulated in different compartments of kernel. Eighty-five percent of the oil is stored in the embryo, whereas 98% of the starch is located in the endosperm [30]. The accumulation patterns of major grain constituents (e.g., starch, oil, and protein) in maize are genotype specific [31]. The protein content was not affected with o-CDs treatments in this study. Yang et al. (2022) [32] showed that protein content in maize grains could be increased by 49.7% after the treatment with nitrogen-doped CDs under drought conditions, but with a reduction in the yield. CDs can also increase the protein content in soybean seeds under drought stress by 3.4% [33].

Photosynthesis is the main source of assimilation for crop yield formation, and the size of yield is determined by the accumulation and distribution of photosynthetic products. As a physiological process capable of converting light energy into a usable chemical energy, photosynthesis involves photosynthetic pigments and photosystems, the electron transport system, and the CO_2_ reduction pathway [34]. The impact of different o-CDs concentrations on photosynthetic parameters has previously been studied in maize plants and green beans cultivated in hydroponics [14,25] and in the soil [15]. The obtained findings of all photosynthetic parameters (Figure 2) are consistent with our initial results obtained from maize plants in a laboratory setting, where we demonstrated the stimulative effects of foliar o-CDs application on photosynthetic intensity and transpiration [14]. Therefore, the stimulative impact of o-CDs is now confirmed under conventional agricultural conditions in an open-field plant trial.

Since photosynthetic pigments are an important parameter of photosynthesis, we intended to investigate the effect of o-CDs on them to understand their connection with grain yield. However, the obtained results regarding o-CDs’ effect on the content of photosynthetic pigments (Figure 3) indicate that the pigment content was not a prevailing reason for the yield increase. The 5 mg/L of o-CDs led to the increased Chla and Chlb in ZP 6263, while the 1 mg/L of o-CDs increased Chlb in ZP 4567. The literature data showed that CDs could significantly increase the expression of chlorophyll enzymes in rice, which helps to improve chlorophyll synthesis and CO_2_ assimilation [35]. Also, enhanced chlorophyll content, by 15.41%, was noticed in the maize after the foliar treatment with CDs [27].

TAA and TPC are indicators of metabolic disorders in plants. TAA includes the contribution of different nonenzymatic components with antioxidant capacity (vitamins, phenolic acids, sugars, etc.), while TPC reflects the contribution of phenolics as a group of secondary metabolites participating in the regulation of plant growth and in defense responses [36]. Since the use of fertilizers that cause plant stress is not recommended, TAA and TPC analysis is of great importance. The obtained results (Figure 4) showed that TAA and TPC were not affected by the different o-CD treatments, so it can be concluded that these nanoparticles can be safely used as nanofertilizers in agriculture.

Computational simulations and spectroscopic measurements, reported in the previous papers, indicated that o-CDs enhance photosynthetic efficiency by acting as a “CO_2_ delivery” system [15,25]. Due to their small size (~2 nm) and amphiphilic properties, o-CDs easily pass through the cell wall pores. This process of the increased CO_2_ supply to the leaves may be the main reason for the correlative increase in photosynthesis and grain yield. Although the grain yield increase was found to be significant only for the first hybrid (Figure 1), the observed stimulation of CO_2_ assimilation measured two days after foliar treatments aligns with the trend of an increase grain yield across all genotypes, despite the high grain yield variability in maternal corn lines.

The effect of some other types of carbon nanoparticles was reported in the literature [37], but data about the effects of the nanoparticles on fruit yield are rare. Li et al. (2016) [38] reported the influence of the used carbon nanoparticles on the morphological properties of the mung bean plants (root and stem length, and mass of fresh plants). The nanoparticles’ concentration was significantly higher than in our experiment. The authors did not grow the plants to fruits, but did the experiment for a short time under controlled conditions. We have grown the plants in the field until they bear fruit and found an increase in fruit yield. Also, the authors stated that the used nanoparticles accumulate in the beans. We have experimentally confirmed that our nanoparticles are not present in fruits [15], which means that they are metabolically degraded, and thus are not toxic to plants. Wang et al. (2018) [39] observed the positive effects of the used carbon nanoparticles, chemically different from those in our experiment, on certain photosynthetic parameters of mung bean; the plant growth, biomass, and carbohydrate content was increased. However, plants were not grown to fruits. Currently available data from the literature about the effects of foliar treatments of different nanoparticles on cereals show that NPK nanoparticle fertilization increased the yield of wheat [40]. Also, the literature is abundant with data on the impact of the foliar application of inorganic nanopartcles on the grain yield increase in different cereals, but these types of nanoparticles are not comparable with organic CDs.

## 5. Conclusions

The presented results show that increased photosynthetic activity in all studied maize varieties, including hybrids and their maternal components, led to a higher grain yield and improved the composition and quality of maize grains. However, only hybrid ZP 4567 showed statistically significant results. The treatment with o-CDs increased the starch content in both the hybrid ZP 4567 and the inbred line L56 L026, while a significant increase in oil content was observed only in the inbred line L56 L026 after the higher o-CDs concentration was applied. The applied nanoparticles appeared to have a more beneficial effect on the bioactive compounds in the leaves, potentially enhancing the plant’s resistance to biotic and abiotic factors, which remains to be explored in our future research. Also, the effect of o-CDs should be investigated in other cereals to compare with the maize results presented in this study. It would also be interesting to investigate the effect of o-CDs on the absorption and utilization efficiency of nutrients such as nitrogen, phosphorus, and potassium.

## Figures and Tables

**Table 1:** Basic chemical composition of maize grain.

		ZP 4567	ZP 6263	L56 L026	L76 B044
Protein	Control	8.30 ± 0.15	8.54 ± 0.27	12.05 ± 0.42	11.02 ± 0.01
	1 mg/L o-CDs	8.61 ± 0.27	8.65 ± 0.37	10.94 ± 0.96	10.14 ± 0.12
	5 mg/L o-CDs	8.26 ± 0.01	9.02 ± 0.06	11.15 ± 0.48	10.59 ± 0.48
Starch	Control	68.61 ± 0.11 ^b^	70.31 ± 0.04 ^ab^	69.02 ± 0.05 ^a^	68.40 ± 0.08 ^b^
	1 mg/L o-CDs	69.17 ± 0.12 ^a^	70.55 ± 0.21 ^a^	67.36 ± 0.09 ^b^	67.64 ± 0.10 ^c^
	5 mg/L o-CDs	69.63 ± 0.10 ^a^	69.87 ± 0.01 ^b^	67.42 ± 0.20 ^b^	69.73 ± 0.22 ^a^
Oil	Control	4.16 ± 0.01	4.46 ± 0.20	3.87 ± 0.14 ^b^	4.47 ± 0.14
	1 mg/L o-CDs	4.44 ± 0.03	4.35 ± 0.03	4.25 ± 0.13 ^ab^	4.30 ± 0.08
	5 mg/L o-CDs	4.10 ± 0.35	4.06 ± 0.17	4.51± 0.04 ^a^	4.24 ± 0.12
Cellulose	Control	2.30 ± 0.03	2.31 ± 0.07	2.41 ± 0.12	2.34 ± 0.33
	1 mg/L o-CDs	2.34 ± 0.04	2.15 ± 0.10	2.18 ± 0.19	2.14 ± 0.23
	5 mg/L o-CDs	2.36 ± 0.15	2.12 ± 0.12	2.32 ± 0.40	2.19 ± 0.01

^abc^ indicates a significant difference between treatments inside the same maize variety (α = 0.05).

## Data Availability

Data is contained within the article or the datasets generated and/or analysed during the current study are available in the [OECD] repository. Available online: https://www.oecd.org/content/dam/oecd/en/topics/policy-sub-issues/seeds/full-variety-list.pdf (accessed on 15 December 2025).
